# Integrated Approach to Facilitate Stakeholder Participation in the Control of Endemic Diseases of Livestock: The Case of Peste Des Petits Ruminants in Mali

**DOI:** 10.3389/fvets.2019.00392

**Published:** 2019-11-19

**Authors:** Michel Mainack Dione, Ibrahima Traoré, Hamidou Kassambara, Ahmadou Nouh Sow, Cheick Oumar Touré, Cheick Abou Kounta Sidibé, Amadou Séry, Awa Sadio Yena, Barbara Wieland, Martin Dakouo, Oumar Diall, Mamadou Niang, Cheick Oumar Fomba, Modibo Traoré, Abdou Fall

**Affiliations:** ^1^International Livestock Research Institute, Animal and Human Heath Program, Herd Health Team, Ouagadougou, Burkina Faso; ^2^Impact at Scale Program, International Livestock Research Institute, Bamako, Mali; ^3^Laboratoire Central Vétérinaire, Bamako, Mali; ^4^International Livestock Research Institute, Animal and Human Heath Program, Herd Health Team, Addis Ababa, Ethiopia; ^5^Independent Consultant, Bamako, Mali; ^6^Direction Nationale des Services Vétérinaires, Bamako, Mali

**Keywords:** small ruminants, PPR, stakeholder, participation, innovation platforms

## Abstract

In Mali, small ruminants (SRs) are an important means for enhanced livelihood through income generation, especially for women and youth. Unfortunately, opportunities for livestock farmers to tap into these resources for economic growth are hindered by high burden of endemic diseases such as peste des petits ruminants (PPR). A key component for the control of PPR is vaccination of SRs. However, low participation of farmers to vaccination was identified by stakeholders of the livestock value chains as a key constraint to successful vaccination programs. This study was implemented in the framework of a project which aimed at improving the domestic ruminant livestock value chains in Mali by upscaling proven interventions in animal health, feeds and feeding and livestock marketing. The objectives of the study were to review the context of livestock vaccination in Mali and evaluate the impact of innovation platforms (IP) as a means for engaging stakeholders in the vaccination process. Desk review, key informant interviews (KII) and net-mapping were used to understand the context of livestock vaccination, while vaccination coverage and sero-monitoring together with group interviews were used to measure the impact of the intervention. IPs were created in 24 communes in three regions: 15 IPs in Sikasso, 4 IPs in Mopti and 5 IPs in Timbuktu. They developed work plans and implemented activities focusing on improving interaction among key vaccine chain delivery stakeholders such as farmers, private veterinarians, vaccine manufacturers, local leaders and public veterinary services; involving them in the planning, implementation and evaluation of vaccination programs and fostering knowledge sharing, communication and capacity building. After 2 years of implementation of IPs, vaccination coverage for SRs increased significantly in target communes. During the first year, seroprevalence rate for PPR increased from 57% (CI95: 54–60%) at baseline to 70% (CI95: 67–73%) post-vaccination in Sikasso region, while in Mopti region, seroprevalence increased from 51% (CI95: 47–55%) at baseline to 57% (CI85: 53–61%) post-vaccination. Stakeholder engagement in the vaccination process through facilitated IPs was successful in fostering participation of farmers to vaccination. However, a sustainable vaccination strategy for Mali would benefit from consolidating the IP model, supported by Government investment to strengthen and adjust the underlying public-private-partnership.

## Introduction

Mali's economy is primarily based on agriculture and agro-pastoralism (1). Livestock farming is the main source of income for over 30% of the population, contributing 15% of the country gross domestic products (2). Small ruminants (SRs) represent a significant part of the livestock sector with ~40 million heads in 2016 (3). However, the development of the livestock sector is constrained by high burden of diseases, with peste des petits ruminants (PPR) being a major production constraint (4). PPR is one of the most widespread, infectious and contagious diseases of sheep and goats, with mortality rates exceeding 90% in immunologically naive populations (5). The disease results in high economic impact (6), thus threatening the food security and sustainable livelihood of farmers (7). Although originally characterized and confined in western Africa in the early part of the twentieth century (8), PPR has since been confirmed throughout most of the African continent, as well as the Middle East, central Asia and eastern China (5, 7, 9). The disease is caused by a morbillivirus, PPR virus (PPRV), closely related to the human pathogen measles virus (MV), as well as other animal pathogens such as canine distemper virus (CDV) and rinderpest virus (RPV) (10). Clinical signs of the disease vary and may include ocular and nasal discharges, fever, tissue necrosis, and in most of the cases death of SR livestock occurs within 10–12 days post-infection (11). Once confirmed, the most effective way to control PPR in a given area is mass immunization of SRs (5). There are many vaccines that are commercially available and have shown to be effective for at least 3 years post-vaccination (11, 12), but most of them require a strict cold chain, which represents a key challenge in resources limited countries with high temperatures such as Mali. Since the main route of transmission of PPR is by direct contact, animal movement control is also effective but is difficult to implement in many of the infected countries where extensive and mobile production systems are common (13). In Mali, PPR control strategies have been mainly based on annual national mass vaccination programs (also called “vaccination campaigns”) and/or focal vaccination in response to overt outbreaks. However, in practice vaccination of the entire SR population is difficult to achieve and is costly. For several decades, efforts have been made by the Government to support vaccination campaigns against PPR. Despite significant improvements made so far, results have not shown satisfactory vaccination coverage across the country. This is usually explained by the low level of participation of farmers to vaccination (14). The situation is a result of a combination of many factors including low awareness of farmers about the benefits of vaccination, poor planning of vaccination campaings, poor communication among the vaccine chain stakeholders, amongst others (14, 15). To increase vaccination coverage, there is need for an innovation that would encourage participation of stakeholders in the delivery of vaccines. Such innovation would put emphasis on knowledge sharing, communication and interaction among stakeholders.

Our research was conducted through a development project that aimed at improving productivity of ruminant livestock in Sikasso, Mopti, and Timbuktu regions of Mali from 2016 to 2019. The project aimed at improving animal health, feeds and feeding systems and farmer's access to market (16). To address animal health aspects, the project focused on ways to increase livestock vaccination coverage especially for SRs. This specific study addresses the question of whether increased awareness, communication and interaction among stakeholders of the vaccine chain delivery through an innovation platform (IP) (17) can trigger participation of farmers to vaccination.

## Conceptual Framework

Livestock vaccination in Mali shares characteristics of complex socio-economical systems given the fact that different stakeholders involved, both private and public, have distinct objectives, capacities and incentives. There is often remarkable lack of interaction among these stakeholders. This situation prevents learning and flow of information between them (14). Decision to adopt a new technology involves critical steps including knowledge (awareness) about the technology, gaining sufficient information on its characteristics, benefits, and costs (18). Thus knowledge and information sharing are important factors that influence technology adoption (19, 20). However, the magnitude of the impact of a technology is determined by the rate of adoption, following the diffusion and learning about the technology or innovation over time (20). An IP approach has huge potential to addressing the organizational constraints of the livestock vaccine chain delivery. The IP framework was developed to provide insights into the complex relationships between the diverse stakeholders including farmers, community leaders, vaccine manufacturers, vaccinators, researchers, livestock traders and other input and service providers. Having been increasingly established within the framework of AR4D initiatives (21), they acknowledge the interdependency of stakeholders to achieve agricultural development outcomes, and hence address the need for a space where they can learn, negotiate and coordinate to overcome challenges and capture opportunities through a facilitated innovation process (17). In the context of livestock vaccination, IPs are used to enhance learning, communication, interaction, coordination, and innovation capacity among mutually dependent (but disconnected) stakeholders with different backgrounds, expertise and interests. Given that stakeholders are more likely to support the implementation and scaling of innovations when they have been involved in the design and testing process (22, 23), IPs promote participation and contribute to use of knowledge as to generate possible solutions in a more practical and effective way. Bearing in mind that the concept of innovation systems to address complex agricultural problems is not new, this study focused on the practical application of the concept in the context of livestock vaccine delivery in Mali.

## Materials and Methods

### Site Selection

**Figure 1:**
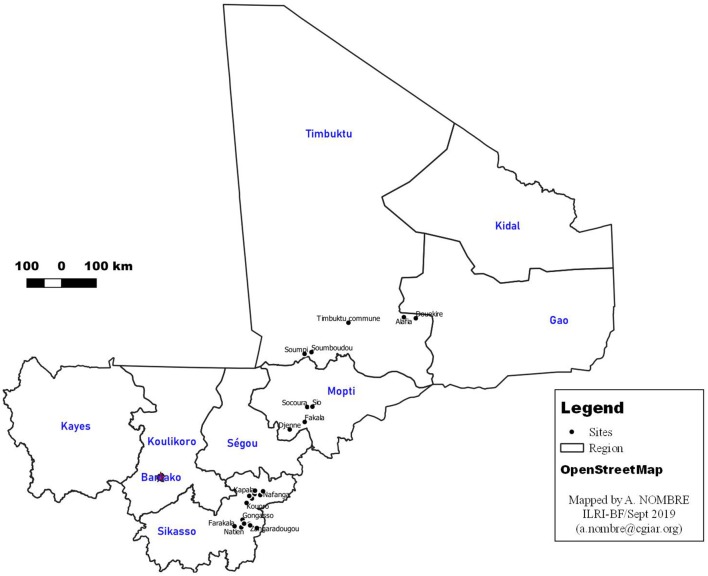
Map of Mali showing areas where the study was carried out.

The study was carried out in three major livestock producing regions of Mali, namely, Mopti and Timbuktu (known as pastoral systems) and Sikasso (known as agropastoral system). The choice of the study area was dictated by the development project that supported the IP activities. In the Mopti and Timbuktu regions, reduced rainfall, overgrazing, expansion of grazing areas in crop land, drying of water points, and wind erosion results in major constraints related to feed availability. Therefore, pastoralists are forced to travel during part of the year to feed their animals. For the specific case of Timbuktu, insecurity is a major concern, making access to remote farmers difficult. In contrast, the Sikasso region is among the wettest areas of Mali with a clear dominance of agriculture over livestock farming. It is a system for which pasture rangeland is the basic diet of animals. Access of farmers to veterinary services is easier in this region, compared to other regions [(24); Figure 1].

### Desk Review

In order to understand the policy and institutional framework in which the livestock vaccination operates in Mali, several key reports related to animal health delivery system were reviewed. They include annual reports of 2016 and 2017 of the National Directorate of the Veterinary Services (DNSV) and the National Directorate of Industry and Animal Production (DNPIA), the OIE country Assessment of Performance of Veterinary Services, the National Strategy Plan for Eradication of PPR, the National One Health Plan (2019–2020) and the Strategic Framework for Economic and Sustainable Development Plan (2019–2023).

### Stakeholder Engagement

This exercise enabled in-depth assessment of the different stakeholders of the vaccination process, their roles, locations, and perceptions about current vaccination strategies.

#### Key Informant Interviews (KII)

KII can help determine not only what people do but why they do it. Such interviews are excellent for documenting people's reasons for their behavior and people's understandings or misunderstanding of issues (25). We used KII to get insights from key high-level stakeholders about the vaccination process. Participants were officially contacted either by emails or by phone calls to be interviewed at their work places. A variety of stakeholders including researchers, policy makers, public and private veterinary services and livestock vaccine manufacturers were interviewed (either the Director /President or any other resource person). The following organizations were consulted: DNSV, National Centre for Animal Health (CNASA), DNPIA, National School of Applied Rural Economics (EIR), Agricultural Market Observatory (OMA), Central Veterinary Laboratory (LCV), Ministry of Livestock and Fisheries, Association of Private Veterinarians (COVEM), National Association of Veterinarians (ANAVEM), Livestock for Growth Development Project (L4G), and development NGOs partnering with the project.

#### Stakeholder Workshop

A workshop was organized at the beginning of the project in 2016, and brought together high-level stakeholders, mostly those that were interviewed during the KII sessions to discuss issues of animal health service delivery in Mali. A special session was held with animal health experts to further discuss keys issues related to vaccination. Recommendations for improvement of vaccination coverage were also provided.

### Context Specific Stakeholder Mapping and System Challenges

Net-Mapping (NM) was carried out to gain more in-depth understanding of the vaccination process tailored to the local context. It also enabled further scrutinization of the mains issues of vaccination from the field and local perspectives. NM was a powerful tool to explore the roles and relations between the different stakeholders on the ground. Drawing on social network approaches (26), the tool is particularly suitable since it can help identify stakeholders and their formal and informal interactions, as well as examine the flows of information from researchers to help determine the pathways of research-based information (27) It uses interviews and maps as the main research method. The NM exercise was carried out by the project team composed of an animal health, capacity development and livestock experts and a MSc student, with assistance from staff of the project implementing partners. In first instance, information gathered from the desk review and KIIs were used to identify broader stakeholders involved in the delivery of animal health services, who were then invited for the NM exercises. Participants were chosen purposively to represent a specific stakeholder group of the vaccine delivery chain. Two NM processes focusing on livestock vaccination were carried out in each region. Twenty-six stakeholders attended the NM exercise in Mopti region, and 19 attended the NM in Sikasso region. The participants were invited to attend a half day workshop facilitated by the researchers and the project partners. The NM process for each group comprised of three steps: identification of the main stakeholders involved in vaccination and their relationships (who does what? why? how? and with who?), determination of the perceived level of influence of the vaccination by different stakeholders (which stakeholder is seen as more important in the process and why?) and identification of constraints and recommendations for improving vaccination campaigns. The NM process was not carried out in Timbuktu as researchers were not able to access the area due to high insecurity.

### Process Development of the Innovation Platforms

**Figure 2:**
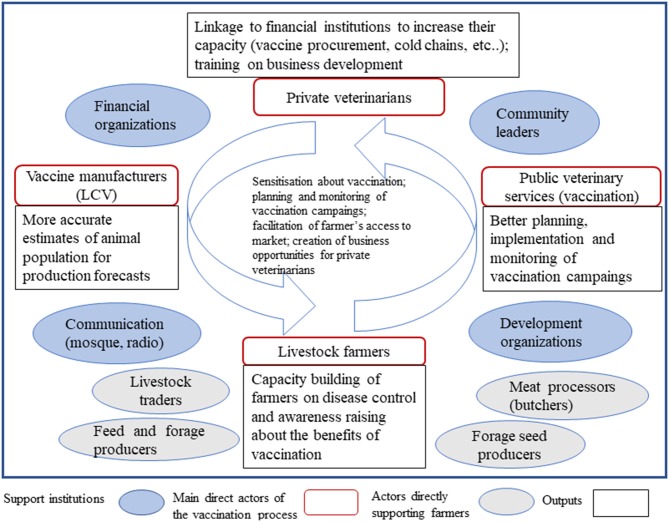
IP structure with types of activities and outcomes.

To establish the IPs, we adopted guidelines as described by Schut et al. (28). IPs were set up in 24 communes of the project: 15 in Sikasso (Natien, Pimperna, Diamatènè, Kafouziela, Zangaradougou, Farakala, Kouoro, Gongasso, Fama, Zangasso, Sinkolo, Kapala, Kolonigué, Nafanga, and N'goutjina); 4 in Mopti (Sio, Djenné, Fakala, and Socoura) and 5 in Timbuktu (Soumpi, Somboudou, Douekire, Alafia, and Timbuktu commune). They were established at the level of a “commune” which is an urban or rural territory collectively acting as a legal administrative entity with financial autonomy. A commune comprises of an average of 32 village with a minimum of 6 villages and a maximum of 58 villages (Table 2). There is a municipal council of elected officials that regulates the economic, social and cultural development affairs of the commune. The project management team held 2 days workshops in each commune to facilitate the creation of the IPs. They were made of representatives of stakeholders identified during the stakeholder mapping namely direct actors involved in the livestock vaccine chain delivery such as farmers, “mandataires,” vaccine producers and public veterinary services; actors directly supporting farmers such as livestock traders, feed stockists and meat processors and institutions supporting the livestock value chain such as financial organizations, community leaders, NGOs, and information systems (Figure 2). Each IP had set up a steering committee comprising at least a coordinator, secretary, treasurer, and communication lead. Representation of women was ensured in each steering committee with at least two positions held by women. IP steering committee members were trained on governance and leadership by the project. Their roles were to convey meetings, develop work plans, document activities, follow up implementation of innovations. During the process of creating the IPs, facilitators identified by the project implementing partners were invited to attend the first meetings and received training in facilitation skills. They were then mentored by the project to ran IP meetings. An IP steering committee met whenever possible (on average once in a month) to review progress of activities, challenges, and opportunities. Capacity development activities were regularly conducted by the project to strengthen technical and organizational capacities of IPs. To ensure adequate documentation of activities and outcomes, monitoring, and evaluation of the IPs followed the project guidelines as advised by the donor. IPs used notebooks to document activities such as meetings and trainings. Project implementing partners draw information and collected data to develop reports sent to the project monitoring and evaluation team at a monthly, quarterly and annual basis. A project planning workshop organized at the beginning of each year during the lifespan of the project allowed interaction between stakeholders, project implementing partners and project core team to discuss successes and issues related to implementation of IPs.

### Impacts Assessment of the IPs

Baseline data on numbers of SRs vaccinated was provided by the “mandataires” in their respective communes and backed up with data obtained from the public veterinary services in each target communes prior the start of the intervention in 2016. The same information was collected after two consecutive vaccination campaigns (2016–2017 and 2017–2018). In addition, a post-vaccination sero-monitoring survey was carried out for the 2016–2017 vaccination campaign, 1 year after the implementation of the IPs. The calculation of the sample size for the sero-monitoring study was based on the recommended 80% sero-prevalence to achieve herd immunity. Blood and serum samples were randomly collected from 1,500 animals before vaccination and the same number starting from 4 months after vaccination. Competitive ELISA was used to measure the level of sero-conversion of animals following vaccination. Laboratory tests were carried out at the LCV in Bamako, Mali.

An evaluation of the status of the IPs was carried out by the project team in 2018, with 15 IPs that responded to the survey. The evaluation team was composed of the project expert on capacity development and implementing partner in each region. An evaluation guideline was developed and administered to a group of two to three IP steering committee members who were selected to be part of the interviews. However, a single response based on group-consensus was recorded. In total, 40 members participated in the interviews. The criteria that were retained for the evaluation were: good understanding of the basic concepts and objectives of the IP by their members; good understanding of the roles of the steering committee; structuration process of the IP in place to see if the process is taking shape; functioning of the IPs and mechanism of self-funding for sustainability.

## Results

### Understanding the Context of Livestock Vaccination in Mali

Currently, disease control in Mali (esp. vaccination) is run through a public-private-partnership (PPP) with farmers largely covering vaccination costs. In the PPP model, private veterinarians hold the sanitary mandate, so they are called “mandataires.” They are supervised by the public veterinary services to implement livestock vaccination in their assigned areas. In areas where private veterinarians are not operating, vaccination campaigns are carried out directly by public veterinary agents. Development organizations support free of charge vaccination in specific areas such as those of high insecurity like Timbuktu region. The objectives of the vaccination campaigns are set by the public veterinary services in consultation with the “mandataires” of each region. The targets (number of animals to be vaccinated during the campaign) depend on the capacities of both public and private veterinary services including availability of funds, equipment and human resources. Several factors that limit performance of vaccination campaigns have been identified in our study. They were groups into three categories: limited participation of farmers to vaccination, limited access of farmers to quality vaccines and socio-economic factors including policy, gender and cultural barriers.

#### Limited Participation of Farmers in Vaccination Campaigns

The high cost of vaccination was pointed out by various stakeholders as being a limitation to farmer's participation to vaccination. Contrary to what is observed for drugs, where costs for SR are below costs for cattle, the cost of vaccination of SR is the same as that for cattle and camel for any disease. This is perceived as not economically sound and psychologically acceptable to farmers who think that it is unfair given the huge difference in value of these animals. Making farmers better understand the purpose and procedures of vaccination was thus seen as necessary. This situation is exacerbated by the packaging size of the vaccine (100 doses per vial) which is not suited to farmers who hold small flock sizes who would require group vaccination to reduce the cost. However, such arrangements (group vaccination) entail additional costs related to farmer mobilization which requires extra time, especially for women, if not well-coordinated. On the other hand, the lack of transparency in the communication of the conditions and side-effects of vaccination was considered by stakeholders as a major concern to farmers. This is caused by the fact that many farmers think that PPR vaccination may result in serious side effects as observed for CBPP vaccine. This situation causes reluctance and fuels the lack of trust between farmers and veterinarians. Therefore, the acceptance of vaccination by farmers will largely depend on their level of awareness about the vaccines used (efficiency and safety). In addition, there is mis-perception about the objectives of vaccination by some farmers who think that vaccination is for fattening animals or for treating already sick animals. This leads to farmers missing opportunity to vaccinate their animals at the right time. Added to that, the poor planning, coordination and evaluation of vaccination campaigns was regarded as a major constraint, causing a fragmented vaccine chain delivery where stakeholders do not have the same information at the same time.

#### Limited Access of Farmers to Vaccines (Quantity and Quality)

Frequent vaccine shortages during the vaccination campaigns have been reported. The inaccurate livestock census prior to vaccination is a major cause for this. Often, animal population statistics provided by veterinary services as a basis for forecasting the vaccine demand are far underestimated because most farmers do not declare all their animals to avoid being taxed, yet the census of animals for vaccination is different to the one for tax collectors, and they are even carried out by different government bodies. In addition, the limited capacities of “mandataires” to stock large quantities of vaccines at required temperatures has raised concerns about the quality of vaccines delivered to farmers. This situation creates a fragile business environment for “mandataires” who need to be supported according to stakeholders. Support to the “mandataires” could be achieved through strengthening their business opportunities by facilitating their access to financial institutions to access loans to purchase equipment and grow their business.

#### Gender and Socio-Cultural Factors

In traditional livestock systems, sheep, goats, and poultry are the main livestock owned and managed by women, who then play an important role in disease prevention and control. The fact that SRs belong to women or are primarily managed by them, especially in sedentary areas, means that men do not feel bothered by their vaccination, so women do not get enough support to participate in vaccination programs. Furthermore, women face time constraints and limited access to information about vaccination schedules. In addition, in most rural communities, women cannot declare ownership of their animals or register themselves for vaccination because they are not recognized as head of the household. For example, during the livestock census, women who own livestock register them under the name of their husband or son. This situation often leads to wrong perception of communities (especially women) that SRs do not need to get vaccinated.

Often factors affecting performance of vaccination programs are present at all levels of the vaccine delivery chain, and they are interlinked and often involve a range of stakeholders at a time. Thus, an integrated participatory process through IP to tackle the main issues seemed a promising approach.

### Stakeholders Involved in the Vaccination Process

The results of the net- mapping revealed a diversity of stakeholders involved in the livestock vaccination process. In both pastoral (Mopti) and agro-pastoral (Sikasso) regions, farmers, vaccine producers, and “mandataires” were perceived as having the greatest level of influence by stakeholders. However, in pastoral area, the decentralized public veterinary services such as Veterinary Sector (SV), Veterinary Post (PV), and the CAHWs held medium level power of influence because they provide vaccination in areas without “mandataires.” In agro-pastoral areas, the central decision-making units such as MEP and DNSV were attributed medium level of power because they are more present. CAHWs scored more in pastoral areas, as compared to agro-pastoral areas. This is probably because in pastoral areas, qualified veterinarians are not readily available. In both areas, the administrative officers, police, and the community leaders scored low. This shows their limited involvement in the vaccination process (Table 1).

**Table 1:** Stakeholders and their level of involvement in the delivery of vaccination.

**Pastoral systems (Mopti)**		**Agro-pastoral systems (Sikasso)**	
**Stakeholder**	**Score**	**Stakeholder**	**Score**
Farmer	9	LCV	11
LCV	7	“Mandataires”	9
“Mandataires”	6.5	Farmer	7
PV	5.5	DNSV	4
Formal drug shop	5	PV	3
SV	4	MEP	2.5
CAHWs	3.5	NGO	2.5
DRSV	3	Administrative officer	2.5
NGO	2.5	SV	2
DNSV	2	CAHWs	2
MEP	1	Community leader	1.5
Ministerial council	0.5	DRSV	1
Police	0.5	DNPIA	1
DNPIA	0	Legal drug shop	1
Community leader	0	Ministerial council	0
Administrative officer	0	Police	0

*During the net-mapping process, participants were asked about their perception of the level of influence of each actor in the livestock vaccination delivery process by stacking small disks according to the level of influence so that the most influential actor has the most stacked disks unlike the least influential actor who has less or none. The allocation of influence scores was set in relation to the delivery of vaccination and not between the actors themselves. To do this, fifty disks were available to all participants, according to their experiences and knowledge, they distributed these discs between different actors. The distribution of influence disks already made was then readjusted if necessary, until the participants were completely satisfied with the degrees of influence attributed. The allocated rank represent an average of two net-mapping exercises per region*.

### Activities Carried Out During the Implementation of the Innovation Platforms

Initially, multiple functions were assigned to IPs besides livestock disease control. However, challenges related to the implementation of vaccination campaigns were considered as a priority to be immediately addressed. Main issues were the poor communication among vaccine chain delivery stakeholders, the poor knowledge of farmers about benefits of vaccination, their low awareness about vaccination schedules, the inappropriate estimation of livestock population for vaccination, the limited implication of women in vaccination and the low capacity of “mandataires.” Each IP developed a yearly work-plan in a participatory manner and carried out the following key activities during each vaccination campaign:

#### Community Census Livestock Population

In each commune, a committee made up of the area “mandataire,” a representative of the IP and a local leader (mayor delegate or village chief) was created to carry out census of SRs prior vaccination to better inform the vaccine demand. Information collected in each village was relayed to the veterinary services and used by the association of “mandataires” to forecast their vaccine stocks with the vaccine manufacturer.

#### Involvement of IPs in the Official Launch of the Vaccination Campaign

Every year an official launching ceremony of the vaccination campaign is organized by the government in one of the communes. During this meeting, the national vaccination calendar and the objectives of the vaccination campaign are communicated. IP members sent a representative to the meeting to get information about these plans. The information is then used to plan sensitization campaigns in their respective communes.

#### Organization of Sensitization/Awareness Campaigns

IPs supported the public veterinary services in organizing awareness campaign about vaccination and dissemination of vaccination calendar through community radio broadcasting in several local languages.

#### Creation of Community Level Committees for the Implementation of Vaccination

IP facilitated the creation of village vaccination teams. The teams were made up of the area “mandataire,” a public authority, a representative of the IP and a local leader. The main roles of the vaccination team were to facilitate the linkage between the community and the vaccinators by setting up the vaccination dates in each village in consultation with the communities and mobilizing farmers for vaccination. Overall, the vaccination team supported the local planning execution, supervision and evaluation of vaccination campaigns together with the veterinary services. Because of insecurity, vaccination teams in Timbuktu region were exclusively composed of CAHWs who are usually supported by GNOs.

#### Capacity Development

IPs facilitated the implementation of capacity development activities for value chain actors on animal health, food safety and livestock production through the promotion of an integrated technological package composed of health, feeding and SR housing training modules.

#### Advocacy for Business Support to “Mandataires”

IPs facilitated linkage between “mandataires” and financial institutions. They supported development of bankable business models through facilitation of trainings of mandataires on business development and management through the project.

Besides vaccination, IPs also discussed and carried out interventions on other topics relevant to them to improve productivity of their livestock such as feeds and feeding, fattening and access markets. Non-specific activities to animal health carried out by IPs to support the livestock value chains include:

#### Support for the Development of Business Models for Livestock Fattening

The main roles of the IPs were to facilitate sheep and cattle fattening activities with an emphasis on promoting group marketing and facilitating linkage between farmers and local and regional markets.

#### Support of the Development of Business Models for Women

IPs foster a conducive environment for women farmer cooperatives to diversify their sources of income through the development and rolling out of viable business models, such as the production and sale of mineral blocks for livestock feeding.

#### Development of a Community-Based Bracharia Seed System to Address Feeding Constraints

IPs, supported model farmers to produce Brachia seed for business. They also mentored farmers to upscale the innovation.

In total, 911 IP events and 495 meetings between IPs and project implementing partners were reported (Table 2). Because of insecurity in the region of Timbuktu, project implementing partners could not join IP meetings.

**Table 2:** Monitoring and evaluation activities of the IPs.

**Region**	**Commune**	**Number of villages**	^*^Number of IP events	^**^Number of meeting between IPs and project implementing partners
Sikasso	Natien	9	39	11
	Pimperna	17	26	9
	Diamatènè	8	18	14
	Kafouziela	7	12	10
	Zangaradougou	7	11	21
	Farakala	12	72	38
	Kouoro	16	68	43
	Gongasso	12	61	37
	Fama	7	27	31
	Zangasso	11	48	31
	Kapala	15	46	20
	Nafaga	6	28	13
	Sinkolo	9	27	22
	Kolonigué	13	42	41
	N'goutjina	8	30	19
Mopti	Sio	35	19	26
	Djenné	16	60	20
	Fakala	46	77	54
	Socoura	58	72	35
Timbuktu	Soumpi	25	27	0
	Somboudou	51	34	0
	Douekire	41	18	0
	Alafia	17	20	0
	Timbuktu commune	8	29	0

* This include IP and community meetings and

** *this include facilitation of IPs and evaluation visits*.

### Outcomes of the Innovation Platforms

#### Increased Vaccination Coverage of SRs

**Figure 3:**
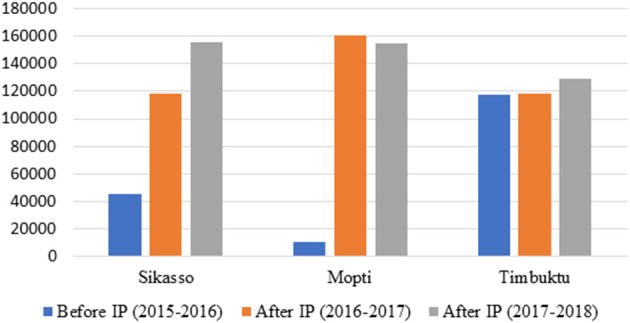
Number of SRs vaccinated in each region before and after establishment of IPs.

Increased participation of farmers to vaccination was shown by the increase in vaccination coverage. Vaccination coverage of SRs has more than doubled over 2 years in target communes compared to previous campaigns (Figure 3). High vaccination rates have been reported in communes of Mopti (Sio, Djenné, and Fakala), Sikasso (Natien), and Timbuktu (Douekire) where vaccination of SRs has never been reported before. In Timbuktu, vaccination is mostly carried out by development NGOs and is free of charge because of insecurity issues. This might explain the lack of noticeable change in vaccination coverage compared to previous years. Also, the monitoring of the IPs was difficult to achieve given that project implementing partners could not directly intervene in this area.

#### Increased Herd Immunity of SRs

**Figure 4:**
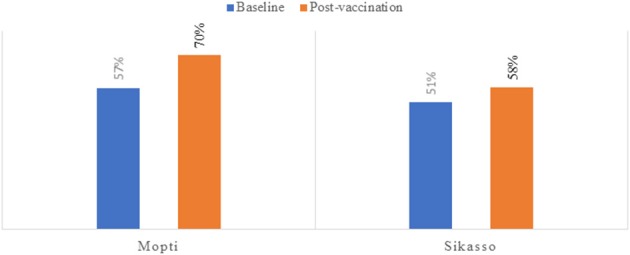
Results of the post-vaccination sero-monitoring of the 2016–2017 vaccination campaign.

Post-vaccination sero-monitoring after 1 year implementation of the IPs revealed an increased sero-prevalence rate for PPR in Mopti and Sikasso regions from 57% (CI95: 54–60%) at baseline to 70% (CI95: 67–73%) post-vaccination, and from 51% (CI95: 47–55%) at baseline to 58% (CI85: 53–61%) post-vaccination, respectively (Figure 4).

#### Performance Assessment of the IPs

**Figure 5:**
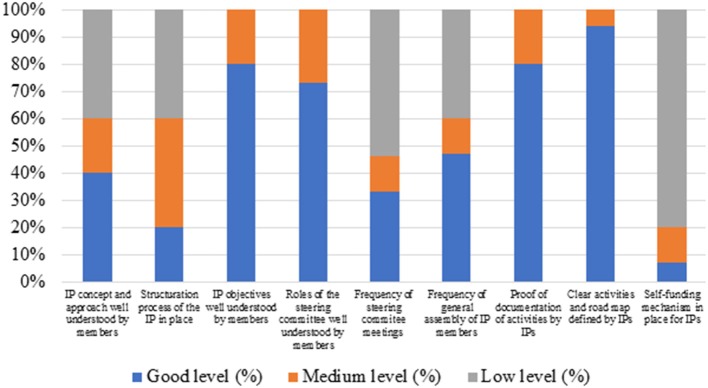
Evaluation of the performance of IPs (*n* = 15) after 2 years of operation.

Assessment of the IPs showed a good understanding of the objectives of the IPs by their members, clear activities, and road map were defined and a good documentation of activities was in place for most IPs. There was however a medium to low level of understanding of the IP concepts by IP members, as well as a medium to low frequency of meetings among the steering committee members. Most IPs did not yet have a sustainable self-funding mechanism in place, so they still rely of project support to run their activities (Figure 5). Major recommendations that emanated from this evaluation include the need for strengthening the endogenous dynamics of IPs and increasing senses of ownership by members; clarifed the terms of references of the steering committees of respective IPs to avoid conflict of interest; reinforce leadership and most importantly intensify the search for self-funding mechanism to ensure sustainability.

## Discussion

### Importance of Stakeholder Engagement in the Vaccination Process

Vaccines are one of the most cost-effective and sometimes the only means to prevent disease in livestock. Commercial vaccines are available for prevention and control of many livestock diseases, however, these vaccines frequently do not reach, and thus are not often used by, smallholder farmers (29). A key challenge to adoption of livestock vaccines in Mali has been the lack of active involvement and limited interaction among stakeholders of the vaccination process (15). In rural sub-saharan Africa, most agricultural development policies have failed to involve stakeholders actively (30). Many studies put the emphasis the importance of stakeholder engagement in livestock disease control (31, 32) but there are few documented case studies. According to Donadeu et al. (29) strategies that could be implemented to increase vaccine adoption should not only consider the use by farmers (access and demand) but also vaccine manufacturing strategies that will ensure adequate vaccine production (availability), because these are the main areas of weakness in the existing vaccine supply chains. The limited involvement of grassroots stakeholders such as livestock farmers and community leaders in the vaccination process was obvious in our project areas, yet these stakeholders were perceived as critical in the vaccine delivery if one wants to reach many farmers with vaccination. This is the reason why an emphasis was put on the participation of local level stakeholder support which is likely to determine disease management success according to Cowie et al. (33). The global strategy for the control and eradication of PPR argues that the true progress in control of PPR and eventually eradication cannot be achieved without serious involvement of relevant stakeholders in all sectors (private and public veterinarians, para-professionals, livestock keepers and their community-based animal health workers, traders, NGOs, and other development partners) (34). Rathod et al. (35) added that global eradication of rinderpest was only possible due to the roles played by all stakeholders, including livestock owners. So, the fact that PPR eradication has been estimated to have the same chances of success as rinderpest, justifies the promotion of approaches that aim at increasing involvement of stakeholders in the control of PPR, hence IP.

The IPs focused on three pillars: knowledge sharing, capacity building and communication. A study in Bolivia and India highlighted the importance of knowledge sharing. The authors concluded that uptake of livestock vaccination was unlikely to improve without knowledge transfer that acknowledges local epistemologies for livestock disease (36, 37). According to Donadeu et al. (29), a good strategy to increase vaccine demand is to increase awareness of the benefits of vaccines and disease control programs. Regular training to livestock owners on vaccination was also suggested in India to boost adoption (38).

The involvement of key stakeholders in all steps of the vaccination process might have contributed to the consolidation of trust among stakeholders, especially between “mandataires” and farmers, resulting in better appreciation of the roles and relations among stakeholders of the vaccine chain delivery. The sensitization campaings that have raised awareness of farmers about the roles and benefits of vaccination might have also motivated farmers to participate in vaccination, hence improvement in vaccination coverage achieved after implementation in the target communes. Although IPs used participatory community approaches for knowledge sharing and dissemination of information, to reach more farmers digital communication channels tools such as interactive voice recording, and text messaging service should be promoted alongside IPs. These are valuable technologies and likely to succeed given the increasing number of farmers who uses mobile phones for business.

### Toward Stronger Public-Private-Partnerships

The PPP in the form of the sanitary mandate is considered by as a suitable approach to control PPR. However, its implementation in Mali has faced many challenges. First, the public good nature of vaccination against diseases such as PPR, that should entail limited vaccine cost to farmers, is contradicted by the current policy of full vaccine cost recovery underway. Resource poor farmers may not see vaccination of their livestock assets as their priority investments, especially if they do not understand the possible long-term benefits. Hence vaccination coverage is below target, which hampers effective disease control. Second, there is an increasing demand from stakeholders to review the legal roles of para-veterinarians and CAHWs who seems to be the only animal health service resource for farmers in areas where qualified veterinarians are absent. In those areas, community initiatives would be a solution to support disease control; and third given the lack of financial incentive in private veterinary practice, many veterinarians have redirected their efforts to other activities in the livestock sector such as production, or even other professions, because current business models are not profitable. Which seems a paradox given the importance of livestock for the country. Therefore, there is imminent need for strengthening PPP and ensure that they are fair.

In the short term the focus should be on finding ways of improving the situation for the already established private veterinarians by strengthening their capacity. This could be achieved through diversification of their activities beyond the sanitary mandate to generate more business opportunities, which could serve as an incentive for them to remain in the job. This could for example be the extension of their mandate to the control of food of animal origin and contribution to epidemiological surveillance or include more activities such as provision of extension services. There is also an urgent need to fill up the current critically low human capacity in the public and private veterinary sector through increasing the number of trained qualified veterinarians and support them in establishing private businesses. This could be achieved by creating a Government support fund for the newly graduated veterinarians. Furthermore, business models that uses private partners such as socio-professional organizations of farmers, economic operators or financial institution for financing vaccination campaigns against major endemic livestock diseases could be tested. In any case, the level of the financial cost contribution of the farmers to vaccination of important endemic diseases such as PPR should be reviewed to ensure these are affordable and fair given that PPR vaccination is considered a public good.

### Sustainability of the Innovation Platform

Agricultural innovation has an important institutional dimension that takes time (39). Ayantunde et al. (40) argue that the performance of IPs seems to improve with the lifespan which underscores the necessity of a long-term perspective for IPs. However, sustainability of IPs will depend on their capacity to generate own funding to run activities. Options for self-financing through private sector actors, such as “mandataires,” are already being promoted by the project, with some IPs pilot testing them. This involves allocation of a percentage of their (“mandataires”) vaccination income to the IPs for their functioning. Other options include diversification of activities of IPs besides animal health. In addition, IPs should be supported with a legal framework that will enable them to be formally recognized by the government irrespective of the form they adopt, either association or cooperative providing it is in line with government regulations. This could help them be well-placed to attract funding from various sources including financial institutions.

In our case, 3 years of implementation was considered short to fully assess sustainably of IPs. However, present achievements provide a basis to capitalize on. Long term monitoring the IPs is necessary to lay solid foundation that will lead to sustainability. Follow up studies will focus on better understanding the social dimensions and dynamics of IPs, to better reveal key drivers for behavioral change of stakeholders.

## Conclusion

Stakeholder involvement in the vaccination process through IP approach has led to an increase of participation of farmers to vaccination, resulting in an increase in vaccination coverage against PPR in target communes. While we promote the upscaling of IPs in other parts of the country, we also call for addressing critical challenges they face in their sustainability pathway. A private business model supported by a solid policy framework is required to sustain such innovation. Although significant progress has been made in increasing vaccination coverage in Mali, the national vaccination coverage is still not enough to guarantee control of PPR anytime soon. A sustainable vaccination strategy will require concerted efforts among stakeholders of the livestock value chains and those of the vaccine delivery, supported by Government investment to strengthen and adjust the PPP models.

## Data Availability Statement

The datasets generated for this study are available on request to the corresponding author.

## Ethics Statement

Ethical approval was not provided for this study on human participants because the activity was implemented with the National Directorate of Veterinary Services (DNSV) in the framework of their national legal mandate under authorization number: N0057/MEP-DNSV. The human subjects were the program beneficiaries. They provided their written informed consent to participate in this study. Ethical review and approval were not required for the animal study because the sero-monitoring activity was carried out by the Central Veterinary Laboratory (LCV) with the approval of the DNSV (reference: N0057/MEP-DNSV) in accordance with their national mandate. Written informed consent for participation was not obtained from the owners of the animals because the sero-monitoring is a routine activity carried under the same approval.

## Author Contributions

AF and MMD conceived the study. MMD compiled the whole information and wrote the manuscript. MMD, IT, HK, ANS, CT, CS, AS, AY, MD, OD, MN, CF, MT, and AF participated in the data collection for key informants and workshops. MMD and AY designed the net mapping tools and collected the data. MMD, IT, and AF designed the questionnaire for the assessment of the innovation platforms. MMD, BW, CS, and AS designed the sero-monitoring study and tools. CS, MD, and AS collected the data for the sero-monitoring and carried out the laboratory analysis. BW, AF, OD, and MMD participated in the structuring and orientation of the write-up. All authors contributed to the literature review performed to build this review, critical review of the manuscript, and approved the final version.

### Conflict of Interest

The authors declare that the research was conducted in the absence of any commercial or financial relationships that could be construed as a potential conflict of interest.
